# Development and Psychometric Testing of Coding Scheme for Dementia Family Communication Using Video Observation

**DOI:** 10.1093/geroni/igab046.2757

**Published:** 2021-12-17

**Authors:** Sohyun Kim

**Affiliations:** University of Iowa, Iowa City, Iowa, United States

## Abstract

Understanding communication behaviors between persons living with dementia and family caregivers is essential for meaningful social interaction and decrease problematic behaviors and caregiving burden. The purpose of this study was to develop and test the psychometric properties of a coding scheme for dementia care interactions. The coding scheme items were developed from literature and expert review, and the pilot testing on 16 video-recorded interactions. A secondary analysis was conducted using 77 videos from 21 dyads of dementia family interactions naturally occurred in the participant’s home. The final coding scheme consists of 11 codes for persons living with dementia (6 nonverbal and 5 verbal) and 12 codes for family caregivers (7 nonverbal and 5 verbal). Content validity was excellent (I-CVI = .93, S-CVI/UA = .71, S-CVI/Ave = .93 with 6 experts). Inter-item correlation was acceptable for both caregiver codes (positive nonverbal = .21, positive verbal = .15, negative nonverbal = .36, negative verbal = .29), and patient codes (positive nonverbal = .13, positive verbal = .27, negative nonverbal = .15, negative verbal = .18). Intra-rater reliability (Cohen’s Kappa = .83, percentage of agreement = 83.88%) and inter-rater reliability (Cohen’s Kappa = .81, percentage of agreement = 81.75%) were excellent. Findings suggest the preliminary psychometric properties of the newly developed coding scheme to assess dyadic interactions of persons living with dementia and their informal caregiver in-home care situations. Future testing of the coding scheme for application in communication interventions to improve quality social interaction in dementia care is discussed.

